# Adverse Moral Selection: Moral Injury and the Moral Composition of the Clinical Workforce

**DOI:** 10.1111/jep.70584

**Published:** 2026-09-08

**Authors:** Boris Nikolic

**Affiliations:** ^1^ Department of Philosophy Heinrich‐Heine‐Universität Düsseldorf Düsseldorf Germany

**Keywords:** clinical workforce, moral distress, moral injury, moral residue, moral responsibility, normalisation of deviance, professional ethics

## Abstract

**Rationale:**

Clinician distress has been progressively reframed over the past decade: from burnout, construed as an individual failure of resilience, to moral injury, construed as a systemic failure that betrays the practitioner's commitment to the patient. The reframing has correctly relocated responsibility from the individual to the institution, but it has stopped at the level of the injured individual and has not traced what injury does to the composition of the profession itself.

**Aims and Objectives:**

This paper takes that further step, asking how the distribution of moral residue within clinical teams affects which practitioners remain in the workforce and which leave.

**Method:**

Conceptual analysis drawing together three established but largely disconnected literatures: moral distress and its accumulation as moral residue, moral injury, and the normalisation of deviance. Results Felt moral residue is allocated across a clinical team not by causal contribution or culpability but by self‐ascribed answerability: the practitioner who construes himself as answerable absorbs the residue, while the practitioner whose omission lay in the causal chain may register nothing. Because accumulated residue drives attrition, and because insensitivity to deviance confers persistence, the institution tends to retain its least morally responsive members and to shed its most responsive. Conscience thus behaves as a trait under negative institutional selection, degrading the moral composition of the workforce as a structural matter and independently of any individual's choices.

**Conclusion:**

On this account, interventions directed at the distress of the conscientious, absent structural change, may accelerate rather than arrest the drift. Implications for professional self‐governance and for the design of clinical institutions are drawn.

## 1 The Reframing and Its Limit

For more than a decade the dominant idiom for clinician suffering was burnout, a syndrome of emotional exhaustion, depersonalisation and diminished accomplishment located in the practitioner and addressed through the practitioner's own resources [1]. The vocabulary carried an implicit aetiology: distress as a depletion of personal resilience, remediable by self‐care, mindfulness and wellness programmes. Its remedies were, accordingly, individual ones.

The reframing of that distress as moral injury was a deliberate correction [2]. Adapted from the study of combat trauma—where the injury follows the betrayal of what is right, by someone holding legitimate authority, in a high‐stakes situation [3]—and given a working psychological model by Litz and colleagues as the lasting harm of perpetrating, failing to prevent or witnessing acts that transgress deeply held moral commitments [4], the concept was imported into medicine to name what burnout misdescribed. Its claim was that the distress is not a defect of the practitioner but a wound inflicted by the system, and that the locus of remedy is therefore institutional rather than individual [2, 5].

That correction was right, and it relocated responsibility correctly. But it stopped at the level of the injured individual. The moral‐injury literature tracks the wound within the wounded, and counts its prevalence across a workforce treated, implicitly, as a fixed roster of practitioners who are each more or less injured. It has not asked the prior question: who remains in that roster, and why? Injury, on the standard account, is something that happens to the members of a population whose membership is taken as given. The membership is not given. It is, this paper argues, an output of the very process that injures.

The argument is as follows. Felt moral residue—the lingering moral sediment of distress [6, 7]—is allocated across a clinical team not by causal contribution to an outcome, nor by culpability for it, but by the practitioner's self‐ascribed answerability. Because accumulated residue drives exit, and because insensitivity to deviance confers persistence, the institution tends over time to retain its least morally responsive members and to shed or numb its most responsive. Conscience, on this account, behaves as a trait under negative institutional selection; the moral composition of the profession drifts accordingly, not through the corruption of any individual but as a structural consequence of how residue is distributed and of what its accumulation does to those who bear it. The argument proceeds from the allocation rule (Sections 2 and 3) to the selection mechanism it generates (Sections 4 and 5), through its defence against counter‐selective forces and objections (Sections 6 and 7), to its consequences for intervention, self‐governance and institutional design (Sections 8 and 9).

## 2 Three Literatures, One Gap

The components of the argument are not new; their assembly is. Three bodies of work, largely developed in isolation from one another, supply the materials, and a precise statement of what each omits locates the contribution.

### 2.1 Moral Distress and Moral Residue

Jameton's coinage fixed the core case: moral distress arises when one knows the ethically right course but is constrained from taking it [8]. Webster and Baylis named the sediment such episodes leave—moral residue, that which one carries from the occasions on which one has compromised oneself or been compromised [6]. Epstein and Hamric modelled the dynamic over time as a crescendo effect: repeated distress raises a baseline of residue that does not fully dissipate and which, accumulating, tends towards one of three terminations—moral numbing, escalating conscientious objection, or burnout and departure [7]. The literature thus already contains, in outline, both the desensitisation and the exit on which the present argument will draw. What it does not contain is any account of how residue is allocated among co‐present agents, or of what its accumulation does to the population rather than to the person.

### 2.2 Moral Injury

The injury literature is distinguished from burnout precisely by its relocation of fault from the individual to the system [2, 5], and is defined structurally by betrayal at the hands of legitimate authority in high‐stakes circumstances [3, 4]. It has begun, moreover, to observe a differential vulnerability: that those most motivated by the patient's good may be the most exposed when systemic constraint prevents them from serving it. The observation is read, however, as a fact about who is wounded, and not as a fact about who subsequently remains.

A closely adjacent literature describes the clinician as a ‘second victim’ of adverse events and records the heightened exposure of the most committed. It names who is wounded among co‐present agents; it does not theorise how residue is allocated among them by answerability, which is the present contribution.

### 2.3 Normalisation of Deviance

Vaughan's analysis of the Challenger disaster described how unsafe practice, repeatedly unpunished by catastrophe, is progressively redefined as normal [9]. Banja transposed the account to healthcare, where egregious departures from standards persist for years, rarely from malice and typically under performance pressure, until they cease to register as wrong [10]. Its psychological engine is moral disengagement, the selective deactivation of self‐censure that allows ordinarily conscientious agents to participate in harm without distress [11].

Set side by side, the three describe a state within individuals (residue), a process upon individuals (injury) and a drift of collective standards (normalisation). None treats the cross‐agent allocation of residue; none treats residue as a selection pressure on a workforce; and, most consequentially, none connects the desensitised survivor of the distress literature with the disengaged functionary of the deviance literature, although they may be the same practitioner observed from two disciplinary vantage points: the affective numbing of the distress literature and the cognitive disengagement of the deviance literature can converge on one behavioural outcome—reduced responsiveness to deviance—by distinct routes, with different antecedents and different prospects of reversal. The remainder of this paper supplies those connections.

## 3 The Allocation Rule: Residue Tracks Self‐Ascribed Answerability

Consider a resuscitation that fails for want of a supply that was never ordered—an omission of a required task of a kind that is both documented and prevalent in clinical settings [12, 13]. The attending who directs the effort competently, and who is, of those present, among the least implicated in the omission, carries the death for days afterwards. The team member whose unmade order lies in the causal chain displays nothing. The distribution of felt residue here is not merely uneven; it runs inverse to causal contribution and orthogonal to fault.

The thesis of this section—advanced as a proposed explanatory principle rather than an empirically established rule—is that felt residue settles according to self‐ascribed answerability (the practitioner's construal of himself as the one answerable for the outcome), and that this construal is keyed to role‐identity rather than to the causal facts. Williams and Nagel established that residue may attach to the authorship of an outcome rather than to blame for it: the lorry driver who, through no fault of his own, kills a child feels—and, Williams argues, appropriately feels—an agent‐regret that the equally careful driver who kills no one does not [14, 15]. But the moral‐luck literature theorises the individual's relation to his own luck‐inflected outcome; it does not address the distribution of such residue across several agents jointly implicated in a single outcome, which is the clinical case.

The responsibility distinctions supply the missing apparatus, and the choice among them is not incidental. Watson distinguished two faces of responsibility: attributability, on which an act or attitude is one's own as an expression of one's evaluative self, and accountability, on which one is liable to the demands, sanctions, and reactive attitudes of others [16]. Shoemaker argued that a third notion lies between them—answerability, the aptness of being called to offer one's reasons, to justify the judgement or quality of will from which one acted [17]. These three come apart, and the clinical case turns on their coming apart. On an attributability reading, residue should settle on the agent whose conduct most expresses a defective quality of will, which, if anyone, is the practitioner who failed to order the supply. On an accountability reading, it should track liability to sanction, which institutional practice tends to assign by proximate cause or by protocol. Neither predicts the observed distribution. What the attending exhibits is the felt correlate of answerability: the sense that the outcome is his to explain, that he is the one of whom an account is apt to be demanded and who demands it first of himself. Residue, on this account, is answerability turned inward—self‐ascribed answerability, felt.

Answerability so understood is indexed to role rather than to cause. The attending construes the resuscitation as his to answer for because the role of the physician running the code carries, in its very self‐understanding, a comprehensive and non‐delegable answerability for the patient before him; the discrete and delegable task of replenishing a supply carries no comparable self‐ascription, even where its omission is causally decisive. The role supplies the standing from which one takes oneself to owe the account, and it is the taking, not the causal contribution, that the residue follows. This is why the distribution inverts: comprehensive answerability attaches to the role least likely, on any given occasion, to have been the proximate cause, while the proximate cause attaches to a task whose holder claims no answerability for the whole.

The rule is not vacuous, for it predicts alignment as readily as inversion. Vary the case: a proceduralist perforates a vessel during an intervention he himself performs and directs. Here causal contribution and self‐ascribed answerability converge in a single agent—the role of the operator carries comprehensive answerability for the procedure, and the operator is also its proximate cause—and the prediction is that residue tracks cause exactly, settling on the one who both acted and answers for the act. It does. Divergence appears precisely where comprehensive role‐answerability and proximate causation fall to different agents, as in the multi‐agent omission, and convergence where they fall to the same one. That a single rule generates both patterns, and predicts which from the structure of the case, is what distinguishes it from the bare observation that distress is unevenly felt.

Several other variables plainly correlate with who suffers—proximity to the patient at the moment of death, seniority, the culture of a given specialty and the gendered distribution of emotional labour among them. The claim is not that these are inert. It is that they operate upstream of the allocation rule rather than as competitors to it: each shapes who is socialised to construe himself as answerable. Proximity furnishes the occasion for self‐ascription; seniority enlarges the answerability a role is taken to carry; professional and gendered norms train some practitioners more than others to internalise the patient's outcome as their own to account for. Self‐ascribed answerability is therefore offered as the proximate variable through which the others exert their influence, not as a rival to them.

Empathy and emotional proximity warrant separate treatment, since they are the most natural rivals to the proposed rule. They cannot, however, explain the distribution. The bedside nurse may be more emotionally proximate to the dying patient than the attending, and may feel the loss more acutely as grief, yet carry less residue where she does not construe herself as ultimately answerable for the outcome; conversely, a physician may carry substantial residue in a case involving little personal attachment, because the role he occupies carries answerability irrespective of feeling. Emotional proximity may intensify residue once it has settled, but it does not fix where it settles. Answerability does. The distinction matters for what follows, for it is answerability, not affect, that is bound to role and therefore liable to the institutional selection the next sections describe.

Two limits should be stated plainly. First, self‐ascribed answerability is itself partly dispositional and partly constructed by the very institution whose composition is in question—a circularity to which Section 6 returns, and which the selection argument in fact requires. Second, the allocation rule is a claim about the central case, not an exceptionless law. The argument needs only the weaker claim that, in the characteristic multi‐agent case, felt residue and causal contribution come systematically apart, and apart in a determinate direction: residue loads onto the role‐answerable rather than the causally proximate. That is the claim the clinical phenomenology supports, and it is sufficient for what follows.

Three levels of support should be distinguished and not conflated: (a) that clinicians experience distress after adverse events is well evidenced; (b) that such distress is associated with intention‐to‐leave and attrition is likewise supported, including by the MMD‐HP literature [18]; and (c) that felt residue is distributed among co‐present agents by role‐based self‐ascription rather than causal involvement—the allocation rule proper—is the present conjecture, established by neither (a) nor (b). The MMD‐HP evidence bears on the exit pathway; it does not reach the cross‐agent allocation.

## 4 From Allocation to Selection: Two Pathways of Depletion

Grant the allocation rule. Its consequences are not confined to the moment of the case. Iterated across careers and aggregated across a workforce, it generates a selection pressure on the moral composition of the profession, carried by two pathways.

The first is exit. The agent who reliably self‐ascribes answerability reliably accumulates residue. The crescendo model supplies the sequel: an accumulating residue tends, at the limit, towards burnout and departure [7]. The moral‐distress literature documents that terminus—practitioners who leave a post or the profession altogether [7, 19]—and the moral‐injury literature documents its more acute form among precisely the most committed [2, 4]. The high‐residue practitioner is, over time, differentially removed from the population.

**Figure 1:**
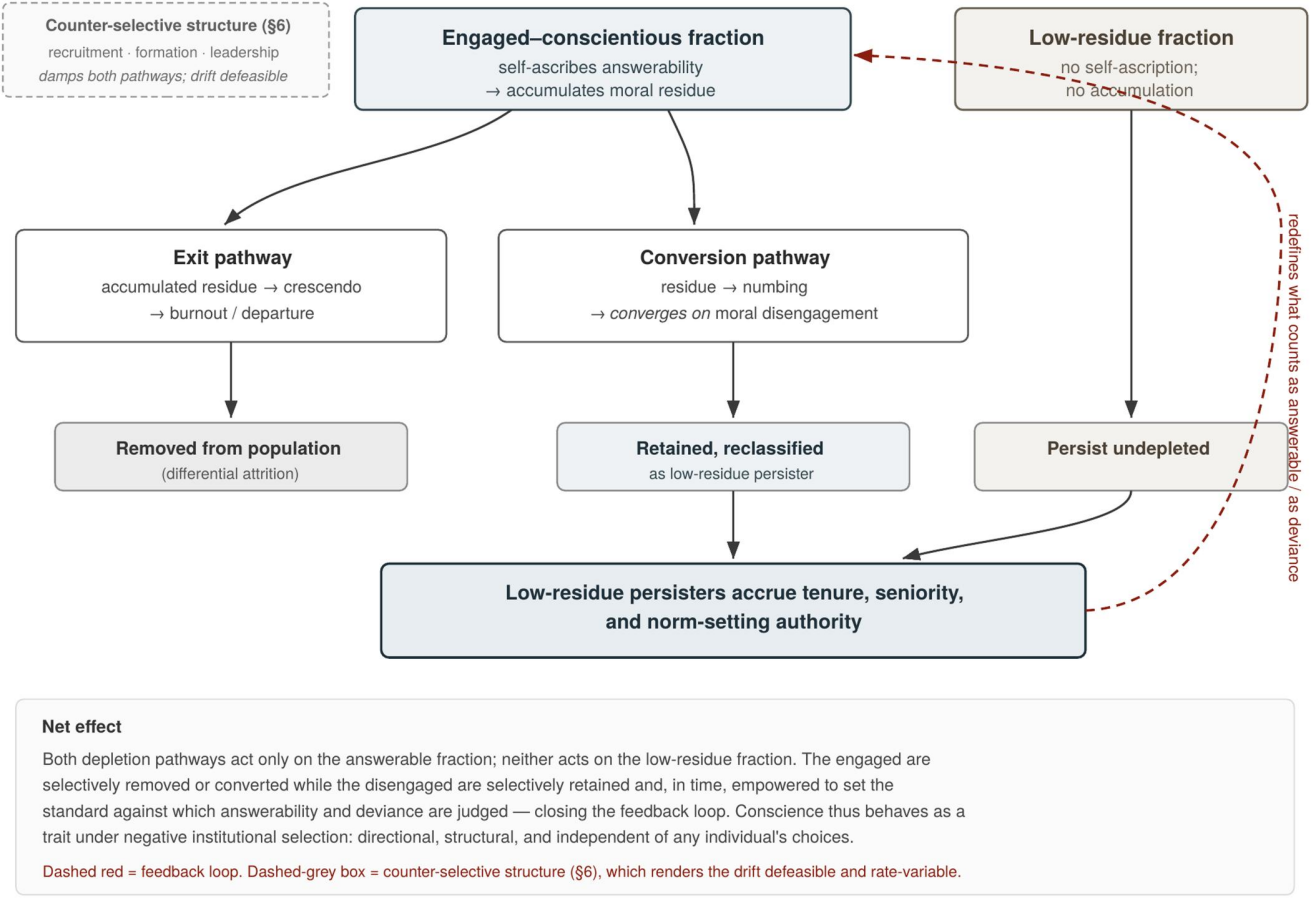
Adverse moral selection as a pressure on workforce composition. The engaged–conscientious fraction—practitioners who self‐ascribe answerability and so accumulate moral residue—is depleted by two pathways: exit (residue → crescendo → burnout and departure) and conversion (residue → numbing, converging on moral disengagement), the latter retaining the practitioner in post while reclassifying him as a low‐residue persister. The low‐residue fraction, self‐ascribing no answerability, persists undepleted. Persisters of both kinds accrue tenure, seniority, and the authority to set local norms, thereby redefining what shall count as answerable and as deviance (dashed feedback). Counter‐selective structure—recruitment, professional formation and enacted leadership—damps both pathways, rendering the drift defeasible and its rate institution‐variable. The net effect is a directional, structural selection against a disposition that the profession exists to require, independent of any individual's choices.

The second pathway is conversion, and it is the hinge of the argument. Departure is not the only way out of the engaged‐conscientious fraction. The crescendo model's first terminus is not exit but numbing—the practitioner who remains in post while his moral sensitivity is progressively extinguished [7]. Described in the vocabulary of the other literature, the numbed survivor is the disengaged functionary of the normalisation account [10, 11]: one who continues to practise while the deviation no longer registers as wrong. The two literatures may be describing convergent endpoints reached by distinct routes. Numbing (an affective desensitisation) can serve as one antecedent pathway into disengagement (a motivated deactivation of self‐censure); what the distress literature sees as the burning‐out of sensitivity and the deviance literature as the settling‐in of normalised deviation meet in a shared behavioural outcome—reduced responsiveness to deviance—rather than being one process under two names. By this pathway the engaged‐conscientious fraction is depleted without anyone leaving, its members reclassified as low‐residue persisters (Figure 1).

Both pathways act on the same fraction—those who self‐ascribe answerability and feel its residue—and neither acts on the low‐residue practitioner, who by construction neither accumulates towards burnout nor possesses the sensitivity that numbing would remove. The low‐residue therefore persist undepleted; and, persisting, they accrue tenure, seniority and the authority to set local norms, including the norms that govern what shall count as answerable and what shall count as deviance. The composition of the workforce drifts accordingly. The proportion of engaged‐conscientious practitioners falls—not because any individual is corrupted, but because the engaged are selectively removed or converted while the disengaged are selectively retained and, in time, empowered to define the standard against which engagement is measured.

The status of this claim must be stated with care, lest it be mistaken for an empirical finding it does not contain. The mechanism is conceptual. Its inputs are independently evidenced: the allocation of residue by answerability, argued in Section 3; the crescendo from residue to numbing or exit [7]; the differential vulnerability of the committed [4]; and the persistence of the disengaged [10]. Its output—a measurable drift in the moral composition of a workforce over time—is a prediction the mechanism generates, not a result it reports. The prediction is testable, and naming the tests is part of taking it seriously: longitudinal measurement of moral sensitivity against tenure within units; differential‐attrition studies keyed to baseline measures of moral distress or residue [7]; and cohort comparison of units differing in the structural variables identified in Section 9.

To move the central prediction from principle to test, consider one study the account licences. A prospective multi‐site cohort would enrol clinicians within defined clinical units and measure, at baseline, moral distress and residue using a validated instrument—the Measure of Moral Distress for Healthcare Professionals supplies one such baseline [18]. Self‐ascribed answerability, being inward, would be indexed through the behavioural proxies already identified: ethics‐consultation seeking, participation in debriefing, and the self‐reported inability to set an outcome down [18]. Units would be followed over a period long enough for compositional change to express itself—on the order of 2–3 years—with two primary outcomes: attrition, resolved into departure from the unit, the role and the profession; and change over time in a validated measure of moral sensitivity. The discriminating contrast is between selective and morally random attrition: adverse moral selection predicts that exit is correlated with baseline residue after adjustment, and that mean moral sensitivity within a unit falls with cohort tenure, whereas ordinary turnover predicts neither. Candidate confounders must be carried explicitly—specialty, seniority, workload and staffing intensity, baseline psychological distress, local labour‐market conditions and organisational ethical climate—since each offers a non‐selective route to the same attrition. A result in which residue predicts exit, and sensitivity declines with tenure, net of these, would separate the drift this paper predicts from the churn with which it must not be confused; a null result would bound the mechanism's reach. The argument therefore stands as a conceptual hypothesis with evidenced premises. It does not, and on the present evidence cannot, assert the drift as established fact.

## 5 Adverse Moral Selection: The Analogy and Its Limit

The mechanism described is a selection effect: directional, structural and independent of the intentions of those it sorts. It is proposed here under the name adverse moral selection.

The novelty claimed lies in the mechanism, not in the phrase: a terminology search returned no prior scholarly use of ‘adverse moral selection’ in this sense. The term is retained because it names, in one compression, a directional and structural selection effect; where the economic resonance misleads, ‘negative moral selection’ or ‘institutional selection against moral responsiveness’ would serve the same sense.

The economic resonance is deliberate and should be qualified at once, since a misread analogy would do more damage than the label does good. The mechanism is not Akerlof's. The market for lemons turns on an informational asymmetry between parties to a transaction: the seller knows a quality the buyer cannot observe, and the unobservable good is driven from the market. No corresponding asymmetry is required here; nothing in the argument turns on anyone misjudging anyone's quality at the point of any transaction.

The nearer analogue is Gresham's law, on which debased coin drives sound coin from circulation—and even this holds in structure rather than in mechanism. What the two share is the perverse result that the item disadvantaged for survival within the system is the item the system most requires: sound coin in the monetary case, morally responsive practitioners in this one. The trait that the profession exists to require is the trait its internal dynamics select against. The vocabulary of economics is thus offered as a description of a dynamic, not as a derivation from a theorem; the argument of Sections 3 and 4 stands without it, and the term earns its place only as a compression of a mechanism already secured on independent grounds. Retained with that qualification, adverse moral selection names what the constituent literatures separately cannot: not that clinicians are injured, nor that standards decay, but that the injuring and the decay are two faces of one pressure bearing on the question of who is permitted to remain a clinician.

## 6 Counter‐Selective Forces and the Limits of the Claim

The claim is of a net directional tendency, not of a determinism, and several forces run against it. Cataloguing them is necessary both for accuracy and because they locate the variance the account must explain. Medicine recruits, and to a degree still selects for, vocational motivation; the inflow is not morally random, and a sufficiently conscientious intake can outpace the drain. Professional formation, mentorship and the moral exemplars a unit contains can cultivate answerability faster than the mechanism erodes it. And where leadership reliably converts answerability into a shared institutional response rather than into private residue—where, in Vaughan's terms, a commitment to safety is enacted rather than merely declared [9]—the exit and conversion pathways are throttled near their source.

These forces render the drift defeasible and its rate institution‐variable. That is not a weakness of the account but its principal empirical purchase, for the model predicts variance. It explains why two units drawing on the same labour market, the same training pipeline and the same external pressures may diverge over years—one retaining its conscientious core, the other hollowing out—and it locates the difference in whether counter‐selective structure is present. A model that predicted uniform decay would be false to the observed heterogeneity of clinical micro‐cultures; one that predicts conditional, structure‐dependent decay is not. The thesis is therefore not that the moral composition of every workforce must degrade, but that, absent deliberate counter‐selective structure, it tends to, and that the tendency is a property of the system rather than a failing of the people within it.

It remains to discharge the circularity noted in Section 3. That the institution partly constructs the self‐ascription on which the mechanism operates does not undermine the account; it completes it. The institution both shapes the distribution of answerability—through recruitment, formation and the norms its persisters set—and then selects among practitioners through the downstream consequences of that distribution. The relation is a feedback loop, not a vicious circle: today's persisters set the answerability norms that constitute tomorrow's engaged fraction, whose depletion in turn renews the persister cohort. The construction of answerability is thus not a rival to the selection model but one of its stages.

## 7 Objections and Replies

Three objections bear directly on the mechanism and must be met before its consequences are drawn.

The first holds that self‐ascribed answerability, being a private and inward matter, is unobservable, so that a selection claim resting upon it is empirically empty. The objection mistakes the epistemic situation. Self‐ascribed answerability is not directly observed, but neither is moral distress, which the field nonetheless measures through validated instruments and behavioural proxies [7, 18]. The proxies are available here too: who debriefs and who does not, who ruminates, who seeks ethics consultation, who scores highly on a measure of moral distress, who reports an outcome as one they cannot put down [18]. The selection prediction is framed over these proxies—differential attrition and numbing keyed to measured residue—and is testable without any direct access to the inward ascription that explains them. A mechanism whose central variable has measurable correlates is a hypothesis, not a vacuity.

The second objection is deflationary: the compositional drift, if it occurs at all, is the product of ordinary turnover and labour economics, and nothing moral need be invoked. But ordinary turnover is, with respect to conscience, morally random; it removes the engaged and the disengaged alike, in proportion to their numbers, and predicts no change in composition. The present claim is precisely that attrition is not random with respect to moral responsiveness—that it is correlated with it, through the residue that responsiveness generates—and a correlation between the disposition and the exit is what converts random turnover into selective turnover. The deflationary reading is therefore not a rival explanation of the same datum; it is the denial that the datum, a shift in composition rather than mere churn, exists at all. Whether it exists is the empirical question Section 4 leaves open, and one the proposed studies are designed to adjudicate.

The third objection inverts the normative sign. Perhaps the practitioners who persist unburdened are the well‐adjusted, and those who accumulate residue are the maladaptive; if so, the selection is adaptive rather than adverse, and the institution is merely shedding a liability. The objection conflates two metrics. On a metric of individual psychological welfare, a disposition that produces less suffering may indeed be the fitter one, and to that extent the persistence of the unburdened is no pathology. But the adversity at issue is not defined relative to individual welfare; it is defined relative to the function the profession exists to discharge. Answerability for the patient is constitutive of the clinical role, not incidental to the practitioner's comfort, and a process that selects against it degrades the profession with respect to its own purpose, however much it may relieve the individuals it spares. This is the content of the Gresham analogy of Section 5: the trait disadvantaged for survival within the system is the trait the system exists to require. That a disposition is costly to its bearer is no argument that its loss is benign to the institution that needs it.

## 8 The Iatrogenic Corollary

If the mechanism holds, a class of well‐intentioned interventions is not merely insufficient but liable to be counterproductive. Three kinds of individual response should be distinguished [2]. (a) Interventions that individualise a structural problem—resilience training and wellness curricula offered as a substitute for changed condition—raise tolerance of the unchanged. (b) Support offered after morally distressing events—debriefing, ethics support—is not in itself objectionable. (c) Interventions that preserve moral agency while changing the conditions that generate distress are the constructive category. The moral‐resilience literature, on its richer construal, names not merely individual coping but the preservation of moral agency, integrity and ethical climate, placing it in (c) rather than (a). The iatrogenic risk below attaches specifically to (a). Two observations follow. First, such measures operate on the absorber while leaving both the allocation rule and the underlying deviation untouched; they may reduce the felt residue of the answerable without redistributing answerability towards cause and without removing the omissions that generate residue in the first place. Second, and more seriously, to the extent that they succeed in keeping the injured in post while doing nothing to restore the conditions of engaged practice, they convert the exit pathway into the conversion pathway, retaining the formerly conscientious in a numbed rather than a departed state. An intervention that reduces attrition without restoring engagement does not arrest the drift; it accelerates it, by enlarging the disengaged remnant that comes to set the unit's norms.

This is not an argument against supporting distressed clinicians, and it must not be mistaken for one. It is an argument against support offered as a substitute for structural change—against treating the symptom in the answerable as though it were the disease. The point in fact extends the moral‐injury literature's own founding move rather than opposing it. That literature insisted that distress was a system problem miscast as an individual deficit [2, 5]; the present account follows the logic one step further than its authors did, to the conclusion that individually targeted remedies, applied to a system‐level selection pressure, will tend to feed the very process they are meant to relieve.

## 9 Implications for Self‐Governance and Institutional Design

The professions claim, and are granted, substantial authority over their own membership—to license, to credential, to promote and to exclude. That authority is exercised on signals, and the present account exposes a hazard in the signals ordinarily relied upon. Where affect is read as a proxy for fitness—where the visibly haunted practitioner is taken to be impaired or culpable, and the unmoved practitioner taken to be sound—the authority to exclude operates in the same direction as the selection pressure and compounds it. The practitioner who feels the residue of a failure he did not cause presents as the problem; the practitioner whose causal omission produced it, and who feels nothing, presents as well. A self‐governing body that misreads the felt residue of answerability as a marker of unfitness will exile its conscience and credential its disengagement under the very procedures meant to protect the standard. The remedy is not to abandon self‐governance but to sever the inference from affect to fitness, and to read the distribution of residue as evidence about the system—about where answerability has been misallocated—rather than as evidence about the individuals who happen to feel it.

If residue is generated by omissions and misallocated by role, two strategies of design follow. The first is to reduce the load. Forcing functions, defaults and clear ownership that prevent the omission do more for the moral composition of a workforce than any after‐the‐fact attribution that merely distributes its guilt. On this view the much‐debated safety apparatus—the checklist, the hard stop, the engineered default—is not, as its humanist critics sometimes charge, a prosthesis substituting for conscience; it is a reducer of residue load, protecting the moral composition of the workforce precisely by removing the failures that would otherwise generate residue and feed the selection. The second strategy is to reallocate answerability towards cause, so that the agent whose action is causally decisive is also the agent institutionally answerable for it, and residue, where generated, falls where it can do corrective rather than merely corrosive work. Both strategies treat the conscientious practitioner's residue not as a private misfortune to be cushioned but as a signal about institutional structure to be acted upon. Vaughan's warning holds throughout: a structural commitment is as easily proclaimed as ignored [9], and a counter‐selective design that exists only on paper selects no differently from no design at all.

## 10 Conclusion

Moral injury, on the account offered here, is not only a wound suffered by individuals but a pressure exerted upon populations. The unit of harm is not exhausted by the injured practitioner; it includes the changing composition of the profession that injury, iterated and aggregated, brings about. The mechanism is assembled from materials already in hand—moral residue and its crescendo, the betrayal structure of injury, the normalisation of deviance and its engine of disengagement—but assembled into a claim that none of them makes alone: that residue allocated by self‐ascribed answerability, rather than by cause, selectively removes and converts the morally responsive while retaining and empowering the disengaged, so that conscience behaves as a trait under negative institutional selection.

If the claim is sound, three consequences follow. The reframing of distress as a system problem must be carried past the injured individual to the selected population. Interventions that comfort the answerable without redistributing answerability or removing its occasions risk accelerating the drift they are intended to arrest. And the profession's authority over its own membership, exercised on the wrong signal, becomes an instrument of the very erosion it is meant to prevent. The argument is offered as a conceptual hypothesis with evidenced premises, and its central prediction—a measurable drift in moral composition, conditional on the absence of counter‐selective structure—is left as an open and answerable empirical question. The first task it sets is not to comfort the conscience that a system still keeps, but to stop selecting it out.

## Funding

The author has nothing to report.

## Ethics Statement

Ethics approval was not required for this work. The article is a conceptual and normative analysis. It involved no human participants, no animals, and no patient data, and reports no empirical study. The clinical situations described are hypothetical and illustrative, and do not describe identifiable individuals or events.

## Conflicts of Interest

The author declares no conflicts of interest.

## Data Availability

Data sharing is not applicable to this article, as no new data were created or analysed in this study.
